# *STXBP1* Variants Associated With Epilepsy With Variable Severity

**DOI:** 10.1002/cns.71093

**Published:** 2026-08-17

**Authors:** Meng Xu, Xuan Guo, Zhi‐kui Wang, Yang Qi, Ting‐ting Ye, Jie Xu, Wei‐wen Deng, Min‐zhi Zhuang, Yi‐wu Shi, Fu Xiong, Xiao‐rong Liu, For the China Epilepsy Gene 1.0 Project

**Affiliations:** ^1^ Department of Neurology, Institute of Neuroscience, Key Laboratory of Neurogenetics and Channelopathies of Guangdong Province and the Ministry of Education of China The Second Affiliated Hospital, Guangzhou Medical University Guangzhou China; ^2^ The Eighth Affiliated Hospital, Southern Medical University (The First People's Hospital of Shunde, Foshan) Foshan China; ^3^ Department of Medical Genetics/Experimental Education/Administration Center, School of Basic Medical Sciences Southern Medical University Guangzhou China; ^4^ Genetic and Metabolic Center Laboratory Maternal and Child Health Hospital of Guangxi Zhuang Autonomous Region Nanning China; ^5^ Hainan Provincial Key Laboratory for Human Reproductive Medicine and Genetic Research & Hainan Provincial Clinical The First Affiliated Hospital of Hainan Medical University, Hainan Medical University Haikou China

**Keywords:** developmental and epileptic encephalopathy, epilepsy, genotype–phenotype correlation, Munc18‐1, *STXBP1*

## Abstract

**Introduction:**

Pathogenic variants in *STXBP1* are a well‐established cause of developmental and epileptic encephalopathy (DEE), whereas their contribution to milder epilepsy phenotypes remains under‐characterized. We sought to delineate *STXBP1* variants across a clinical severity spectrum and to explore molecular correlates of phenotypic heterogeneity.

**Methods:**

Trio‐based whole‐exome sequencing was performed in patients with unexplained epilepsy recruited from multiple epilepsy centers. Variants were confirmed by Sanger sequencing and interpreted using ACMG criteria. Missense variants were assessed using structural modeling and biophysical prediction tools, and their effects on Munc18‐1 expression were evaluated by immunoblotting in transfected HEK293T cells.

**Results:**

Eight *STXBP1* variants were identified in eight unrelated cases, including one truncating variant (Lys63Ter) and seven missense variants. Five *de novo* missense variants (Pro187Leu, Arg190Gln, Ala251Thr, Arg292His, and Gly544Val) and one *de novo* truncating variant were observed exclusively in individuals with DEE. In contrast, Gly236Val segregated in a family with epilepsy with febrile seizures plus, and Pro434Leu was inherited in a family with self‐limited epilepsy with centrotemporal spikes with incomplete penetrance. *In silico* analyses suggested that Gly236Val and Pro434Leu had comparatively milder destabilizing effects and were located at more solvent‐accessible positions than DEE‐associated variants. Consistently, immunoblotting showed markedly reduced Munc18‐1 levels for DEE‐associated missense variants, whereas Gly236Val and Pro434Leu preserved similar Munc18‐1 levels to wild‐type expression.

**Conclusions:**

*STXBP1* variants may underlie epilepsies of variable severity, extending from milder epilepsy syndromes to DEE. Clinical severity broadly tracks with the predicted structural/biophysical impact of the variant and Munc18‐1 abundance, providing a mechanistic clue for phenotypic heterogeneity in *STXBP1*‐related disorders.

## Graphical abstract

*STXBP1*‐related disorders range from mild epilepsy to DEE, and severity aligns with impact on structure and Munc18‐1 abundance. Noncore inherited variants are stable with preserved Munc18‐1 and a favorable prognosis, whereas de novo core variants reduce Munc18‐1, causing severe seizures, neurodevelopmental deficits, and poorer outcomes.

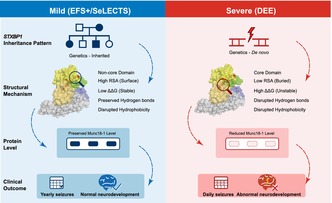

## 1 Introduction

The *STXBP1* gene (OMIM *602926), located at 9q34.11, encodes syntaxin‐binding protein 1 (Munc18‐1), a highly conserved presynaptic regulator of synaptic vesicle fusion. Munc18‐1 binds syntaxin‐1 and coordinates assembly of the soluble N‐ethylmaleimide–sensitive factor attachment protein receptor (SNARE) complex, thereby enabling neurotransmitter release [1]. Disruption of this synaptic machinery leads to a spectrum of neurodevelopmental disorders collectively termed SNAREopathies [2], encompassing epileptic encephalopathies, intellectual disability, autism spectrum disorder, and movement disorders [2, 3]. Among SNAREopathies, *STXBP1* variants represent a major genetic etiology, with over 400 documented cases of *STXBP1*‐encephalopathy [4]. The phenotype is typically characterized by early‐onset intractable seizures, profound intellectual disability, and severe developmental delay. Given the devastating prognosis, affected individuals rarely reproduce, resulting in predominantly *de novo* inheritance patterns. To date, only two cases of asymptomatic parental mosaicism and two homozygous inherited cases have been reported globally [5, 6, 7]. Critically, no mild phenotypes have been conclusively established, creating a significant knowledge gap in understanding genotype–phenotype correlations.

Here, we performed trio‐based whole‐exome sequencing (WES) in patients with unexplained epilepsy and identified eight *STXBP1* variants, including two inherited variants associated with milder epilepsy syndromes. We combined structural/biophysical analyses with cell‐based expression assays to investigate molecular features that may contribute to clinical heterogeneity.

## 2 Methods

### 2.1 Participants and Clinical Phenotyping

Participants were recruited through the China Epilepsy Gene 1.0 Project. Clinical data were obtained for probands and available relatives, including seizure onset age, seizure types and frequency, treatment response, developmental history, neurological examination findings, and family history. Brain MRI was performed to exclude structural lesions. All participants underwent at least one 24‐h video‐EEG recording. Epilepsy syndromes were classified according to International League Against Epilepsy (ILAE) recommendations [8, 9, 10, 11]. A minimum outpatient follow‐up period of 12 months was required.

### 2.2 WES and Genetic Analysis

Genomic DNA was isolated from peripheral blood obtained from probands and their parents/relatives using the Qiagen FlexiGene DNA Kit (Qiagen, Hilden, Germany). We employed the Illumina NextSeq 2000 system (Illumina, San Diego, CA, USA) to conduct trio‐based WES. Sequence alignment, variant calling, and annotation followed previously published pipelines [12]. Candidate variants were confirmed by Sanger sequencing and described using HGVS nomenclature on the NM_003165 transcript. *In silico* pathogenicity prediction used SIFT, PolyPhen‐2, LRT, MutationTaster, DANN, and ClinPred. Evolutionary conservation at variant positions was evaluated through multispecies sequence alignment. Population allele frequencies were queried in Genome Aggregation Database (gnomAD). Variant classification followed ACMG criteria with phenotype correlation and segregation evidence considered where applicable.

### 2.3 Structural and Biophysical Analyses

Structural analyses were performed using the crystal structure of Munc18‐1 (RCSB Protein Data Bank entry 3c98, chain A) as a template. Three‐dimensional visualization and *in silico* saturation mutagenesis were conducted using PyMOL software (v2.5.7; Schrödinger LLC) to assess atomic‐level structural perturbations induced by missense variants. To quantify the impact of these variants on protein stability, the change in Gibbs free energy of folding (ΔΔ*G* or DDG) was calculated using the neural network‐based algorithm I‐Mutant 3.0. All calculations were performed under physiological conditions (pH 7.0 ± 0.5, 25°C ± 2°C). Variants were classified based on thermodynamic stability: destabilizing (ΔΔ*G* < 0 kcal/mol) and stabilizing (ΔΔ*G* > 0 kcal/mol). Solvent accessibility analysis was performed using E‐pRSA [13], a protein language model‐based prediction tool, to calculate sequence‐derived relative solvent accessibility (RSA) values. Residues were classified as buried (RSA < 20%) or exposed (RSA ≥ 20%) based on solvent‐accessible surface area characteristics, reflecting critical features for assessing protein folding energetics and structural stability [14]. The distances between variant residues and their nearest binding partners, as well as the impact of missense variants on protein–protein interaction (PPI) affinity, were computationally predicted using mCSM‐PPI2 [15]. The impact of each amino acid substitution on local hydrophobicity was evaluated based on the Fauchère and Pliska scale [16].

### 2.4 Plasmids, Cell Culture, and Immunoblotting

HEK‐293T cells were acquired from the Cell Bank of the Chinese Academy of Sciences (Shanghai, China) and maintained in Dulbecco's modified Eagle's medium (DMEM; Hyclone, USA) supplemented with 10% fetal bovine serum (FBS; Biological Industries, Israel). Cultures were kept at 37°C in a 5% CO_2_ humidified incubator. First‐strand cDNA was generated using the HiScript II 1st Strand cDNA Synthesis Kit (Vazyme, Jiangsu, China). The full‐length human *STXBP1* coding region was amplified from cDNA and cloned into the pEGFP‐N1 vector (GENEWIZ) via EcoRI and BamHI restriction sites (New England Biolabs, Beijing, China). The cloned *STXBP1* coding sequence was verified by agarose gel electrophoresis, showing a DNA fragment of the expected size (Figure S1A). Site‐directed mutagenesis was employed to introduce specific variants into the wild‐type (WT) *STXBP1* backbone (Table S1). All WT and mutant constructs were confirmed by Sanger sequencing (Figure S1B). Transfections were carried out using polyetherimide (PEI) following standard manufacturer protocols.

Cell lysates were prepared using radioimmunoprecipitation assay (RIPA) buffer (Beyotime Biotechnology). Proteins were resolved by SDS‐PAGE and transferred onto polyvinylidene fluoride (PVDF) membranes. After blocking with 5% nonfat milk, membranes were probed overnight at 4°C with primary antibodies against Munc18‐1 (1:1000; Sigma, HPA008209) and β‐actin (1:10,000; Proteintech, 66009‐1‐Ig). Subsequently, membranes were incubated with HRP‐conjugated goat anti‐mouse secondary antibodies (1:10,000; Proteintech, A00001‐1), and bands were detected using enhanced chemiluminescence.

### 2.5 Genotype–Phenotype Correlation Analysis

To investigate potential genotype–phenotype correlations, the pathogenic impact of missense variants was systematically evaluated using a multiparametric approach. Consistent with recently established classification frameworks in Epilepsia [17, 18], we stratified the variants based on six objective criteria: (1) localization within functionally critical domains (Domains 1 or 3a); (2) disruption of hydrogen bond networks; (3) substantial changes in protein stability (ΔΔ*G* ≥ 0.5 kcal/mol or ≤ −0.5 kcal/mol); (4) alterations in hydrophobicity profiles; (5) RSA < 20% (buried); and (6) evidence of reduced protein expression levels. To further assess the generalizability of these six objective criteria, five representative reported *STXBP1* variants spanning different clinical severities and functional domains were selected for scoring using the same framework. All of these variants have been functionally characterized in the previous studies [7, 19].

### 2.6 Statistical Analysis

Statistical analyses were performed using GraphPad Prism (version 10.1.2). Data normality was verified via the Shapiro–Wilk test. For immunoblot densitometry, values were normalized to β‐actin, and the resulting normalized values were used for statistical comparisons with WT. Group differences were analyzed by one‐way ANOVA followed by Dunnett's multiple comparisons test versus WT. Data are presented as mean ± SEM. All tests were two‐sided, and adjust *P* < 0.05 was considered statistically significant. *n* = 4 independent transfections per group.

## 3 Results

### 3.1 Identification of *STXBP1* Variants

**FIGURE 1:**
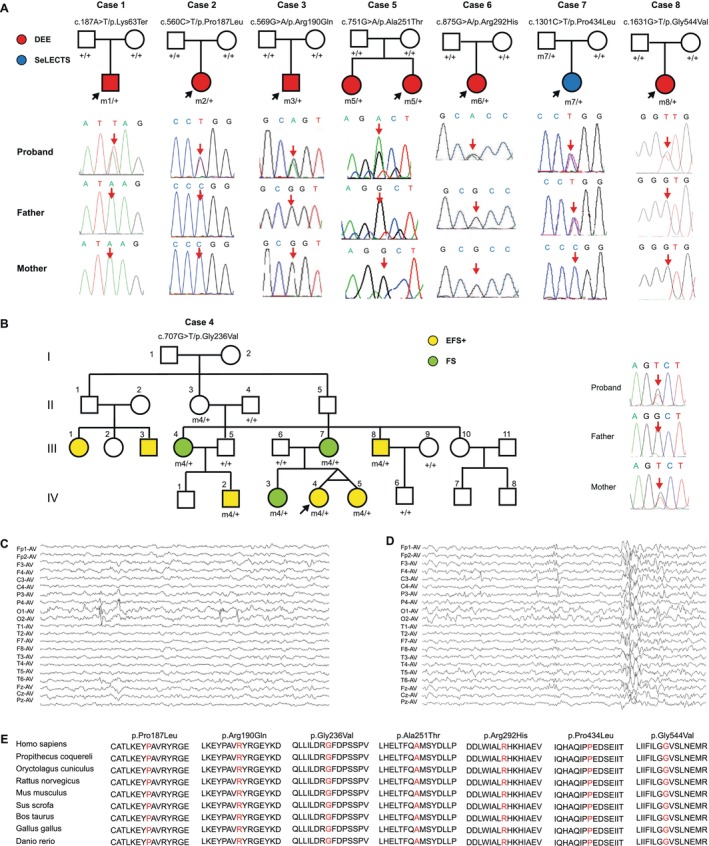
Genetic and clinical features of patients with *STXBP1* variants. (A, B) Pedigrees and Sanger sequencing of *STXBP1* variants in the eight cases with epilepsy. DEE, developmental and epileptic encephalopathies; EFS+, epilepsy with febrile seizures plus; FS, febrile seizure; SeLECTS, self‐limited epilepsy with centrotemporal spikes. (C) Interictal EEG of individual IV‐4 in the family of Case 4 displays bilateral asynchronous spike–wave discharges in central and parietal regions. (D) Interictal EEG of IV‐5 in the family of Case 4 reveals focal and generalized spike‐and‐wave discharges. (E) The amino acid sequence alignment of the seven missense variants shows that the residues are highly conserved across species.

Eight *STXBP1* variants were identified across eight unrelated probands/families (Table 1, Figure 1A,B), including one truncated variant (c.187A>T/p.Lys63Ter) and seven missense variants (c.560C>T/p.Pro187Leu, c.569G>A/p.Arg190Gln, c.707G>T/p.Gly236Val, c.751G>A/p.Ala251Thr, c.875G>A/p.Arg292His, c.1301C>T/p.Pro434Leu, c.1631G>T/p.Gly544Val). Among these, Gly236Val and Pro434Leu were novel variants. Six variants (Cases 1–3, 5, 6, and 8) were identified in patients with developmental and epileptic encephalopathy (DEE), including one familial DEE case involving phenotypically concordant twins (Case 5). Variant Gly236Val was identified in Case 4, a family with epilepsy with febrile seizures plus (EFS+) and demonstrated familial co‐segregation (LOD score = 3.91). Variant Pro434Leu was identified in Case 7, a proband with self‐limited epilepsy with centrotemporal spikes (SeLECTS), and was inherited from his asymptomatic father. Variant Pro434Leu exhibited an extremely low allele frequency (9.147 × 10^−6^) in gnomAD, whereas all other variants were de novo and absent from gnomAD populations. The nonsense variant Lys63Ter was predicted to result in truncated Munc18‐1 protein, leading to a pathogenic loss‐of‐function through haploinsufficiency. All identified missense variants occurred at evolutionarily conserved residues across multiple species (Figure 1E). Furthermore, each identified variant was predicted to be damaging, deleterious, or pathogenic by at least three in silico prediction algorithms (Table 2). According to the American College of Medical Genetics and Genomics (ACMG) guidelines, six out of the eight variants were classified as pathogenic, Gly236Val as likely pathogenic, and Pro434Leu as a variant of uncertain significance (VUS).

**TABLE 1:** Clinical features of the patients with *STXBP1* variants.

Case	Variants	Gender	Inheritance	Diagnosis	Seizure onset	Seizure type	Seizure frequency	EEG	MRI	DD	Response to ASMs
Case 1	c.187A>T, p.Lys63Ter	Male	De novo	DEE (West syndrome)	8 days	Tonic, spasm	10/day	Hypsarrhythmia	Normal	Severe	Resistant
Case 2	c.560C>T, p.Pro187Leu	Female	De novo	DEE (LGS)	2 years	Tonic, GTCS	4/day	GSW, spike rhythm	Normal	Severe	Resistant
Case 3	c.569G>A, p.Arg190Gln	Male	De novo	DEE	1 years + 9months	CPS, sGTCS	6/day	FSW, GSW	Normal	Mild	Controlled
Case 4 (IV‐4, proband)	c.707G>T, p.Gly236Val	Female	Maternal	EFS+	3 years	GTCS, CPS	11/years	FSW	Normal	No	Controlled
Case 4 (IV‐5)	c.707G>T, p.Gly236Val	Female	Maternal	EFS+	3 years	GTCS	7/years	FSW	Normal	No	Controlled
Case 4 (IV‐2)	c.707G>T, p.Gly236Val	Male	Maternal	EFS+	1 year + 5 months	GTCS, CPS	4/years	Normal	Normal	No	Controlled
Case 5 (I‐2, proband)	c.751G>A, p.Ala251Thr	Female	De novo	DEE	1 year + 2 months	Spasm, GTCS	2/day	NA	Normal	Mild	Controlled
Case 5 (I‐1)	c.751G>A, p.Ala251Thr	Female	De novo	DEE	1 year + 2 months	Spasm, CPS, GTCS	3/day	NA	Normal	Mild	Controlled
Case 6	c.875G>A, p.Arg292His	Female	De novo	DEE (West syndrome)	2 months	Spasm	5/day	Hypsarrhythmia	Normal	Severe	Resistant
Case 7	c.1301C>T, p.Pro434Leu	Female	Paternal	SeLECTS	5 years	CPS	2/years	CTS	Normal	No	Controlled
Case 8	c.1631G>T, p.Gly544Val	Female	De novo	DEE (Ohtahara syndrome)	3 days	Tonic, Spasm	5/day	Hypsarrhythmia	Normal	Severe	Resistant

Abbreviations: ASM, anti‐seizure medication; CPS, complex partial seizure; CTS, centro‐temporal spikes; DD, development disorder; DEE, develpomental and epileptic encephalopathy; EEG, electroencephalogram; EFS+, epilepsy with febrile seizure plus; FSW, focal spike wave; GSW, generalized spike wave; GTCS, generalized tonic–clonic seizure; MRI, magnetic resonance imaging; NA, not available; SeLECTS, self‐limited epilepsy with centrotemporal spikes; sGTCS, secondary generalized tonic–clonic seizure.

**TABLE 2:** Genetic features and in silico prediction of *STXBP1* variants.

Variants	SIFT	LRT	Mutation‐Taster	DANN	ClinPred	MAF	ACMG scoring	ACMG pathogenicity	RSA	Protein stability (kcal/mol)	Distance from closest partner (Å)	ΔΔ*G* ^Affifinity^ (kcal/mol)
c.187A>T, p.Lys63Ter	—	D (0)	D (1)	D (0.996)	—	—	PVS1 + PS2 + PM2	Pathogenic	—	—	—	—
c.560C>T, p.Pro187Leu	D (0)	D (0)	D (1)	D (0.999)	P (0.99965858)	—	PS1+ PS2 + PM2 + PP3 + PP5	Pathogenic	0.08	−0.50	17.36	−0.296
c.569G>A, p.Arg190Gln	D (0.005)	D (0)	D (1)	D (0.999)	P (0.99830639)	—	PS1+ PS2 + PM2 + PM5+ PP3 + PP5	Pathogenic	0.15	−0.98	25.12	−0.668
c.707G>T, p.Gly236Val	D (0.002)	D (0)	D (1)	D (0.998)	P (0.99278330)	—	PM2 + PP1 + PP3	Likely pathogenic	0.19	−0.38	26.15	−0.271
c.751G>A, p.Ala251Thr	D (0)	D (0)	D (1)	D (0.999)	P (0.99739193)	—	PS1+ PS2 + PM2 + PP1 + PP3 + PP5	Pathogenic	0.04	−0.68	16.07	0.563
c.875G>A, p.Arg292His	D (0)	D (0)	D (1)	D (0.999)	P (0.99723523)	—	PS2 + PM1 + PM2 + PP3 + PP5	Pathogenic	0.12	−1.14	13.05	0.134
c.1301C>T, p.Pro434Leu	T (0.182)	D (0)	D (1)	D (0.995)	P (0.86377429)	9.147 × 10^−6^	PM2 + PP3	VUS	0.62	−0.05	29.68	0.068
c.1631G>T, p.Gly544Val	D (0.001)	D (0)	D (1)	D (0.998)	P (0.99853670)	—	PS2 + PM1 + PM2 + PP3 + PP5	Pathogenic	0.12	−0.50	17.11	−0.72

Abbreviations: C, conserved; D, damaging/deleterious; MAF, minor allele frequency; P, pathogenic; PM, moderate pathogenicity; PP, supporting pathogenicity; PS, strong pathogenicity; PVS, very strong pathogenicity; RSA, relative solvent accessibility; VUS, variant of uncertain significance.

### 3.2 Clinical Features of Patients With *STXBP1* Variants

The clinical characteristics of patients with *STXBP1* variants are summarized in Table 1. Among the eight probands with *STXBP1* variants in our cohort, six were diagnosed with DEE, including two with West syndrome (Cases 1 and 6), one with Lennox–Gastaut syndrome (Case 2), one with Ohtahara syndrome (Case 8), and two with DEE without a specific syndromic diagnosis (Cases 3 and 5). Age of seizure onset ranged from 3 days to 2 years, with a median onset age of 8 months. All patients exhibited developmental impairment of variable severity. Case 3 and the two affected twins in Case 5 achieved seizure control with combination anti‐seizure medication (ASM) therapy. The remaining cases were poorly controlled with multiple ASMs.

In addition to the DEE phenotypes summarized above, two variants were associated with milder epilepsy phenotypes. Case 4 with Gly236Val was a familial case with epilepsy with febrile seizure plus (EFS+). The variant was co‐segregating across nine affected family members (Figure 1B). Seizure phenotypes in this family included febrile seizure (FS), afebrile generalized tonic‐clonic seizure (GTCS), and focal seizures. Both the proband (IV‐4) and her affected twin sister (IV‐5) showed EEG abnormalities, including focal and generalized epileptiform discharges (Figure 1C,D). All affected individuals had normal cognition and neurodevelopment, unremarkable brain MRI findings, and favorable seizure outcomes. Case 7 with variant Pro434Leu was diagnosed with SeLECTS. The patient presented with sleep‐related focal seizures, with or without evolution to bilateral tonic–clonic seizures. No family history of epilepsy or febrile seizures existed. Neurodevelopment and cognition were normal. Interictal EEG showed sleep‐activated bilateral centrotemporal spikes on a normal background. Seizure freedom was achieved with levetiracetam.

### 3.3 Effect of the Missense Variants on Molecular Structure

**FIGURE 2:**
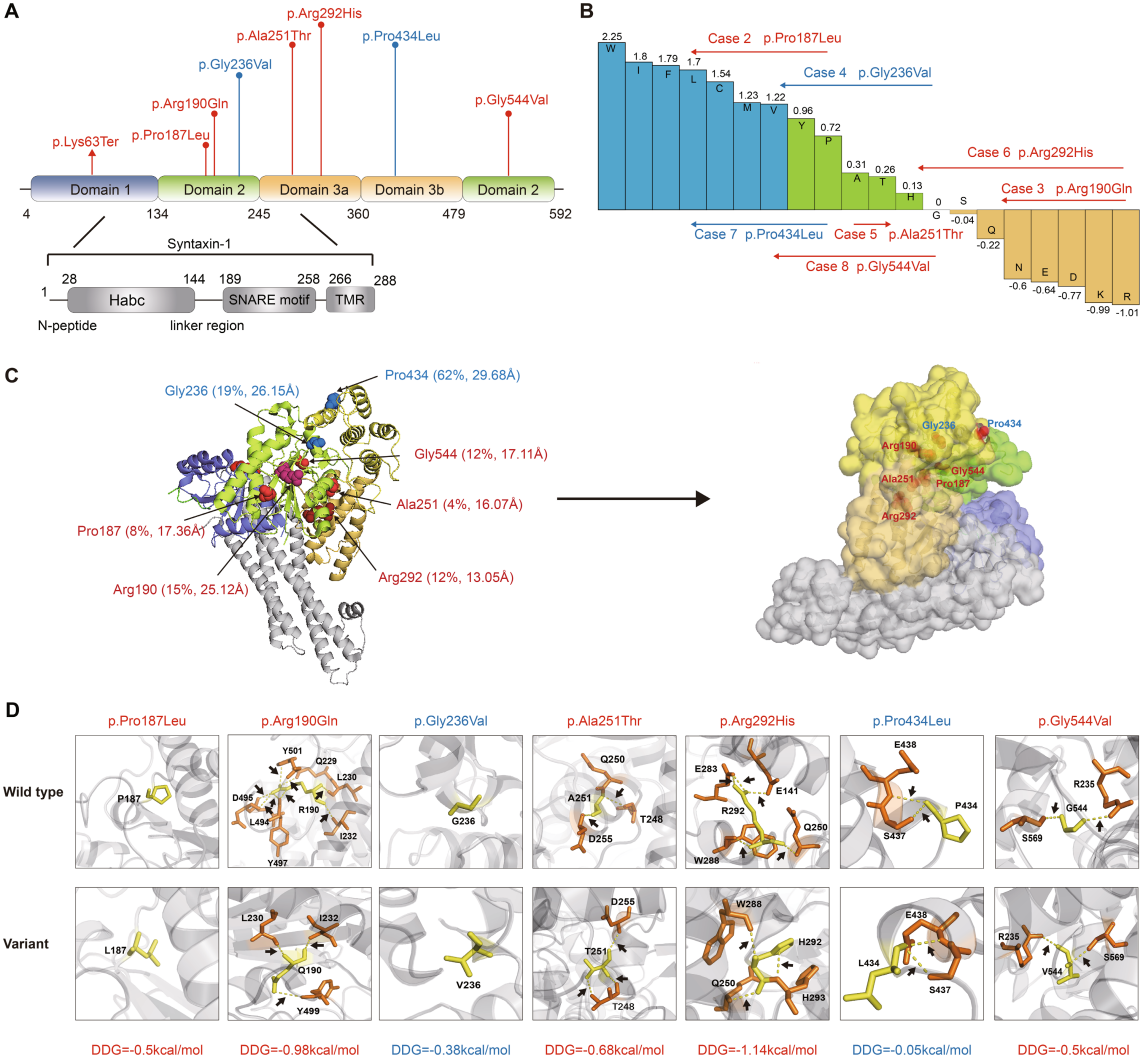
Molecular location, damage effect, and genotype–phenotype relationship of *STXBP1* variants. (A) Domain organization of Munc18‐1 and variant localization. Pathogenic *STXBP1* variants were mapped onto the Munc18‐1 structure, with red highlighting variants associated with developmental epileptic encephalopathy (severe phenotype) and blue highlighting variants associated with milder epileptic disorders. Critical interaction interfaces (Domain‐1 and Domain‐3a) with Syntaxin‐1 are annotated. (B) Fauchère and Pliska's hydrophobicity scale illustrated the hydrophobic characteristics of 20 amino acids. Hydrophobicity decreases progressively from left to right. Hydrophobic amino acids are represented in blue columns, neutral ones in green, and hydrophilic amino acids in yellow. Amino acids with high positive values are more hydrophobic, while those with low negative values are more hydrophilic. (C) Landscape of solvent accessibility for *STXBP1* variants and their distances to the nearest binding partners. The surface‐rendered 3D structure categorizes residues by relative solvent accessibility (RSA) as buried (RSA < 20%) or exposed (RSA ≥ 20%). Studied variants are represented as spheres. (D) Impact of pathogenic *STXBP1* variants on hydrogen bonding and protein stability. Variants with significant destabilization (DDG ≤ −0.5 kcal/mol) or disrupted hydrogen bonding are highlighted in red. DDG < 0 indicates decreased protein stability, while DDG > 0 suggests increased stability.

Munc18‐1 comprises 594 amino acids organized into three distinct structural domains: Domain 1 (residues 4–134), Domain 2 (residues 135–245 and 480–592), and Domain 3 (residues 246–479), with Domain 3 further subdivided into Subdomains 3a (residues 246–360) and 3b (residues 361–479) (Figure 2A). The truncating variant Lys63Ter is mapped to Domain 1. Missense variants are distributed across Domains 2 and 3; Pro187Leu, Arg190Gln, Gly236Val, and Gly544Val are localized to Domain 2, while Ala251Thr and Arg292His are situated in Domain 3a, and Pro434Leu is found in Domain 3b.

Molecular effects of missense variants were assessed through protein modeling in PyMOL. As shown in Figure 2D, variants Arg190Gln, Ala251Thr, and Arg292His altered hydrogen bonding networks with adjacent residues, potentially disrupting overall protein structure. The Arg190Gln substitution reduced hydrogen bonding from eight to three interactions, whereas Ala251Thr generated an additional hydrogen bond with Thr248. The Arg292His substitution abolished two hydrogen bonds; in contrast, the variants Pro187Leu, Gly236Val, Pro434Leu, and Gly544Val preserved WT hydrogen bonding patterns. No hydrogen bonding was observed between residue Pro187 and its surrounding residues in either the WT protein or the Pro187Leu substituted structure; a similar absence of hydrogen bonding was noted for the variant Gly236Val.

Protein stability predictions revealed that Gly236Val and Pro434Leu conferred mild destabilization, whereas Pro187Leu, Arg190Gln, Ala251Thr, Arg292His, and Gly544Val significantly reduced protein stability (Table 2 and Figure 2D). Consistent with their milder stability impact, solvent accessibility calculations revealed that mild epilepsies‐associated variants Gly236 and Pro434 were more solvent‐exposed than the residues affected by DEE‐associated variants (Table 2 and Figure 2C). Five substitutions (Pro187Leu, Gly236Val, Arg292His, Pro434Leu, and Gly544Val) were predicted to alter hydrophobicity based on the Fauchère and Pliska scale (Figure 2B). mCSM‐PPI2 predictions (Figure 2C, Table 2) revealed that Gly236Val and Pro434Leu were in closer proximity to their nearest binding partners than the other DEE‐associated variant residues; nevertheless, the predicted effects of these variants on protein–protein interaction (PPI) affinity did not correlate with their respective phenotypes.

### 3.4 The Decrease of Munc18‐1 Expression

**FIGURE 3:**
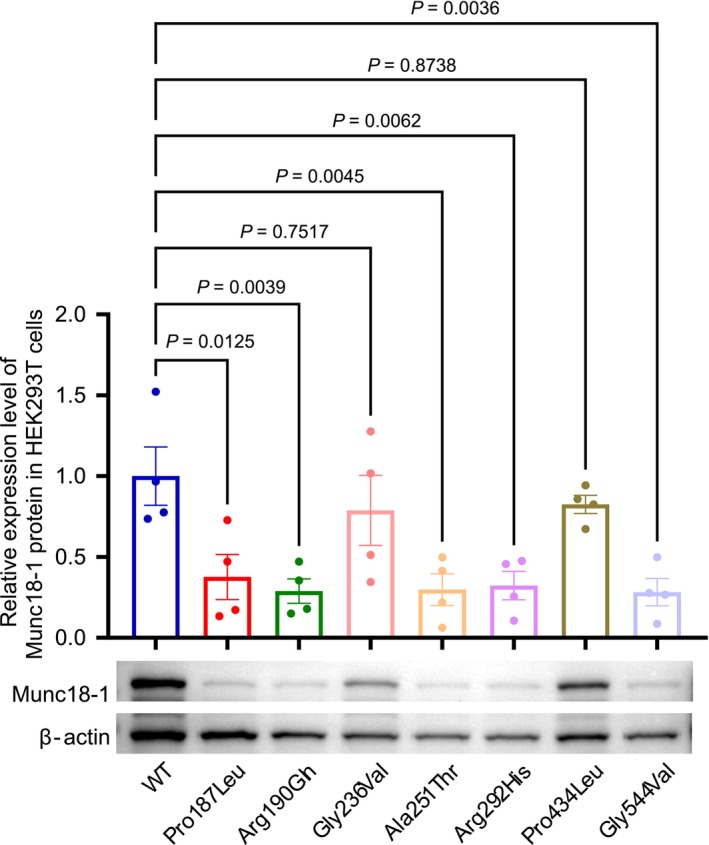
The impact of variants on Munc18‐1 expression detecting by western blotting. Bar plot showed the results of western blot analysis of Munc18‐1 protein levels in WT and mutant Munc18‐1 in HEK 293T cells. Densitometry values were normalized to β‐actin. Data are mean ± SEM (*n* = 4 independent transfections per group). Statistics: one‐way ANOVA with Dunnett's multiple comparisons test versus WT (two‐sided).

To evaluate the functional consequences of seven *STXBP1* missense variants on protein expression, mutant recombinant overexpression vectors were generated by PCR‐based mutagenesis and validated by Sanger sequencing (Figure S1). Western blot analysis in transfected HEK293T cells demonstrated significantly reduced Munc18‐1 protein expression for five variants (Pro187Leu, Arg190Gln, Ala251Thr, Arg292His, and Gly544Val) compared to WT. Although Gly236Val and Pro434Leu also exhibited decreased expression, these reductions did not reach statistical significance (Figure 3).

### 3.5 The Genotype–Phenotype Relationship of *STXBP1* Variants

**FIGURE 4:**
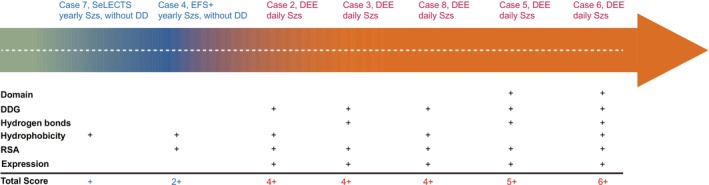
The phenotypic spectrum and genotype–phenotype relationship of *STXBP1* variants. DD, developmental disorder; DDG, the change in Gibbs free energy of folding; DEE, developmental and epileptic encephalopathy; EFS+, epilepsy with febrile seizure plus; RSA, relative solvent accessibility; SeLECTS, self‐limited epilepsy with centrotemporal spikes; Szs, seizures. Scoring: Domain 1 or Domain 3a, +; hydrogen bond alteration, +; significant changes in protein stability, +; alterations in hydrophobicity profiles, +; residue classified as buried, +; markedly reduced protein expression levels, +.

The damage effects of *STXBP1* variants for each case are scored from six aspects (domain, DDG, hydrogen bonds, hydrophobicity, RSA, expression) (Figure 4), which showed a correlation with phenotypic severity. The milder cases (Case 7 with SeLECTS and Case 4 with EFS+) received scores of one and two, respectively. In contrast, the cases with DEE (Cases 2, 3, 5, 6, and 8) received scores ranging from four to six. To external validate the correlation of damage scores and phenotypic severity, the representative reported variants were selected for evaluation. The previously reported asymptomatic variant Leu446Phe received a score of 3. In contrast, the reported variants associated with DEE, including Val84Asp, Cys180Tyr, Met443Arg, and Gly544Asp, received scores ranging from 4 to 6. These findings were consistent with the phenotype–score relationship observed in our cohort (Table S2). These results indicate a potential association between the degree of molecular damage and the severity of the phenotype.

## 4 Discussion

Encoded by *STXBP1*, Munc18‐1 is a brain‐enriched protein of the Sec1/Munc18 (SM) family that plays an indispensable role in synaptic transmission through its interaction with the SNARE complex. The absence of Munc18‐1 results in a complete loss of synaptic transmission [20]. Deletion of *STXBP1* in knockout mice leads to a complete loss of synaptic transmissions throughout development and massive neuron apoptosis after initial synaptogenesis, resulting in widespread neurodegeneration and neonatal death [20, 21]. *De novo* *STXBP1* variants were initially identified in patients with early infantile epileptic encephalopathy (also known as Ohtahara syndrome), a severe neonatal epilepsy characterized by refractory seizures and developmental delay [22, 23]. Transition from Ohtahara syndrome to West syndrome was also observed in patients with *STXBP1* mutations [4, 24]. Subsequent studies extended the phenotypic spectrum to include West syndrome [25], Lennox–Gastaut syndrome [26], Dravet syndrome [27], early myoclonic encephalopathy [28], and other forms of unclassified early‐onset epileptic encephalopathy [29, 30]. Further broadening of the clinical spectrum—encompassing intellectual disability without epilepsy [31], atypical Rett syndrome [32, 33], ataxia‐tremor‐retardation syndrome without epilepsy [34]—established that *STXBP1*‐associated disease extends beyond epileptic encephalopathies, collectively referred to as “*STXBP1* encephalopathy (*STXBP1*‐E)”.

Notably, a reported case of homozygous Leu446Phe manifesting Lennox–Gastaut syndrome, while heterozygous siblings and the mother remained asymptomatic, highlights a critical gap in understanding the full phenotypic spectrum. The three largest cohort studies to date consistently report neurodevelopmental impairment as a near‐universal feature [4, 35, 36], with sample sizes ranging from 32 to 534 patients. The sole individual with borderline intellect carried a somatic mosaic variant and was explicitly excluded from summary statistics as atypical [35]. Crucially, no prior study has reported a patient with a typical heterozygous pathogenic *STXBP1* variant presenting with epilepsy alone and completely normal cognition and development. This gap may partly reflect ascertainment bias, as noted in prior studies [35], where recruitment was skewed toward more severely affected individuals and patients with mild epilepsy and normal cognition were substantially less likely to undergo genetic testing. The increasing accessibility of whole‐exome and whole‐genome sequencing is now enabling identification of *STXBP1* variants across a broader phenotypic range. In this study, we identified eight *STXBP1* variants in patients with epilepsy, including six associated with DEE and two associated with milder phenotypes—one with SeLECTS and one with EFS+—suggesting that *STXBP1* variants may be associated with a clinical continuum ranging from benign epilepsies to DEE.

In this study, the truncated variant Lys63Ter resulted in the premature termination of protein translation and leads to DEE. Five missense variants (Pro187Leu, Arg190Gln, Ala251Thr, Arg292His, and Gly544Val) demonstrated pathogenic links to *STXBP1* encephalopathy, corroborating prior evidence of loss‐of‐function mechanisms of Munc18‐1 [4, 37, 38, 39, 40, 41, 42]. The novel variant Gly236Val was identified in a family with EFS+. The LOD score of linkage analysis provided strong genetic evidence supporting its pathogenicity in this family. Indeed, Munc18‐1 is essential for presynaptic vesicle release by binding syntaxin‐1 and promoting SNARE complex assembly, and it also serves as a molecular chaperone to ensure proper folding, trafficking, and stability of syntaxin‐1 [43]. Misfolding of Munc18‐1 reduces its thermal stability at physiological temperatures and impairs its binding to syntaxin‐1 [22, 44]. Variants in *STX1B* (encoding syntaxin‐1 protein) are associated with epilepsies with variable severity, ranging from focal epilepsy, generalized epilepsy with febrile seizures plus, genetic generalized epilepsy, to DEE [45, 46], which raises the possibility that pathogenic variants in *STXBP1*, engaging in a tight, high‐affinity partnership with syntaxin‐1, may give rise to a wide phenotypic spectrum as well. In addition, our study also identified the novel *STXBP1* variant Pro434Leu with ultrarare population frequency in a SeLECTS pedigree. Although Pro434Leu currently meets ACMG criteria for classification as a VUS, its occurrence in a patient with SeLECTS does not exclude a possible contribution to disease susceptibility. In fact, it is the common feature for the causative genes of milder epilepsies that the variants were rated as VUS, but presented mild pathogenicity and incomplete penetrance, such as *GRIN2A, PGM3, RYR2* [47, 48, 49]. Computational analyses and hydrophobicity analysis supported its potential pathogenicity, while Western blot analysis revealed preserved protein expression, which may explain the attenuated phenotype and the incomplete penetrance observed in the asymptomatic father. A similar phenomenon of incomplete penetrance has previously been described in the variant Leu446Phe. SeLECTS is reported to follow an autosomal dominant inheritance pattern with incomplete penetrance and age dependency [50, 51]. Genetic heterogeneity of SeLECTS involving *ELP4*, *BDNF*, *KCNQ2*, *KCNQ3*, *DEPDC5*, *RBFOX1/3*, *GABAA‐R*, and *GRIN2A* has been documented [52]. Notably, the majority of these causative genes are associated not only with SeLECTS but also with DEE, frequently exhibiting incomplete penetrance. Our findings nominate *STXBP1* as a novel candidate gene for SeLECTS, and the partial functional retention observed here suggests that modifier gene interactions or compensatory mechanisms may contribute to phenotypic attenuation, warranting further investigation.

Previous studies have established that *STXBP1* encephalopathy arises from structural disruption and destabilization of Munc18‐1, resulting in protein aggregation and degradation. These findings identify protein instability and reduced expression as core pathogenic mechanisms underlying *STXBP1* variant pathogenicity [19, 53]. Consistent with this framework, our functional experiments revealed a clear association between Munc18‐1 protein levels and clinical severity: the mild‐phenotype variants Gly236Val and Pro434Leu were associated with only moderate reductions in protein expression relative to wild‐type, whereas all DEE‐associated variants produced markedly diminished protein levels. These findings support a dosage‐dependent model in which the degree of Munc18‐1 deficiency correlates with phenotypic severity.

In structural bioinformatics, RSA is a key determinant of protein folding and stability, with disease‐associated missense variants frequently enriched at buried, structurally constrained positions rather than solvent‐exposed surfaces [14, 54]. Consistent with this principle, the residues affected by Gly236Val and Pro434Leu are significantly more solvent‐accessible than those harboring the five DEE‐associated variants identified in this study. These observations support a genotype–structure–phenotype relationship in which substitutions at evolutionarily conserved, buried residues are linked to more severe clinical outcomes, whereas variants in surface‐exposed loops or solvent‐accessible domains generally correspond to milder phenotypes.

Mounting evidence indicates that the spatial distribution of pathogenic variants within a protein correlates with clinical severity [55, 56, 57]. The interaction between Munc18‐1 and syntaxin‐1 is principally mediated by Domains 1 and 3a [43, 58, 59, 60], which together form an arch‐shaped cleft that envelops syntaxin‐1 [60]. Specifically, Domain 1 engages the N‐terminal peptide of syntaxin‐1 [43], while an extended hinge‐loop in Domain 3a templates SNARE complex assembly [61]. Collectively, these domains constitute the primary interface for syntaxin‐1 binding and are thus central to the regulation of SNARE‐mediated fusion. In this study, the mild‐phenotype variants Gly236Val and Pro434Leu map to Domains 2 and 3b, respectively—regions spatially segregated from the critical syntaxin‐1 binding interface. This structural segregation aligns with the attenuated clinical severity observed in these patients. The similar phenomenon was validated in the reported variants. Variants Ala251Thr and Arg292His in Domain 3a associated with DEE. In contrast, the variant Leu446Phe in Domain 3b—rather than the critical Domains 1 or 3a—manifests exclusively in homozygous individuals, while heterozygous carriers remain clinically unaffected. Functional studies in Munc18‐1‐null neurons demonstrated that this variant causes a comparatively modest reduction in protein stability relative to established pathogenic variants, providing strong support for molecular subregional effects within Munc18‐1.

This study has several limitations, which should be addressed in future investigation. First, the cohort size was modest and skewed toward severe phenotypes, with only one EFS+ family and one SeLECTS case representing the mild end of the spectrum; accordingly, the role of *STXBP1* variants in milder epilepsy phenotypes requires validation in larger cohorts. Second, in this study, functional validation was limited to expression analyses in HEK293T cells, without investigation of variant effects in neuronal systems or on neuronal‐relevant processes such as protein localization, synaptic function, and excitability. Third, our six‐parameter framework should be regarded as exploratory, as the relative contribution and optimal weighting of individual parameters have not yet been established or validated in large datasets. Finally, the Pro434Leu variant remains classified as VUS under ACMG guidelines due to discordant evidence between computational predictions and population frequency data. This raises the demand for developing more sophisticated functional assays to better understand the mechanisms underlying variable penetrance in mild *STXBP1*‐related phenotypes.

## 5 Conclusion

In summary, this study identified six *de novo* variants associated with *STXBP1* encephalopathy and two novel inherited missense variants linked to mild epilepsy with favorable prognosis, hereby expanding the phenotypic spectrum of *STXBP1*‐related disorders. Phenotypic severity for *STXBP1* variants correlated with the degree of structural destabilization, protein expression dose effects, and subregional functional impact, providing potential mechanistic clues to explain the clinical heterogeneity.

## Funding

This work was supported by the National Natural Science Foundation of China (Nos. 82471473, 32570722, 32370649), the Basic and Applied Basic Research Foundation of Guangdong Province (Nos. 2025A1515012437), and the Guangzhou Municipal Education Bureau (Nos. Q23153034). The funders had no role in study design, data collection, data analysis, interpretation, or the decision to publish.

## Ethics Statement

The Ethics Committee of the Second Affiliated Hospital of Guangzhou Medical University provided ethics approval for this study (approval ethics numbers: 2020‐hs‐49). Written informed consent was obtained from the individuals or their legal guardians. The studies adhered to the guidelines of the International Committee of Medical Journal Editors with respect to patient consent for research or participation.

## Conflicts of Interest

The authors declare no conflicts of interest.

## Supporting information


**Figure S1:** Cloning of the *STXBP1* coding sequence and validation of variant constructs. (A) Agarose gel electrophoresis of the cloned *STXBP1* coding sequence (CDS), showing a band at the expected size of approximately 1785 bp. (B) Representative Sanger sequencing chromatograms of WT and mutant constructs. Red arrows indicate the nucleotide substitutions.

**Table S1:** Missense variants were introduced into wild‐type *STXBP1* vectors by PCR‐based site‐directed mutagenesis.

**Table S2:** Evaluation of previously reported *STXBP1* variants using the proposed scoring framework.

## Data Availability

The data that support the findings of this study are available from the corresponding author upon reasonable request.
