# Supplementary material for: STXBP1 Variants Associated With Epilepsy With Variable Severity
Source: CNS Neurosci Ther. 2026 Aug 17;32(8):e71093. doi: 10.1002/cns.71093 (PMC13482176; doi:10.1002/cns.71093)
Supplement: Supplementary file 1 [file CNS-32-e71093-s003.pdf]

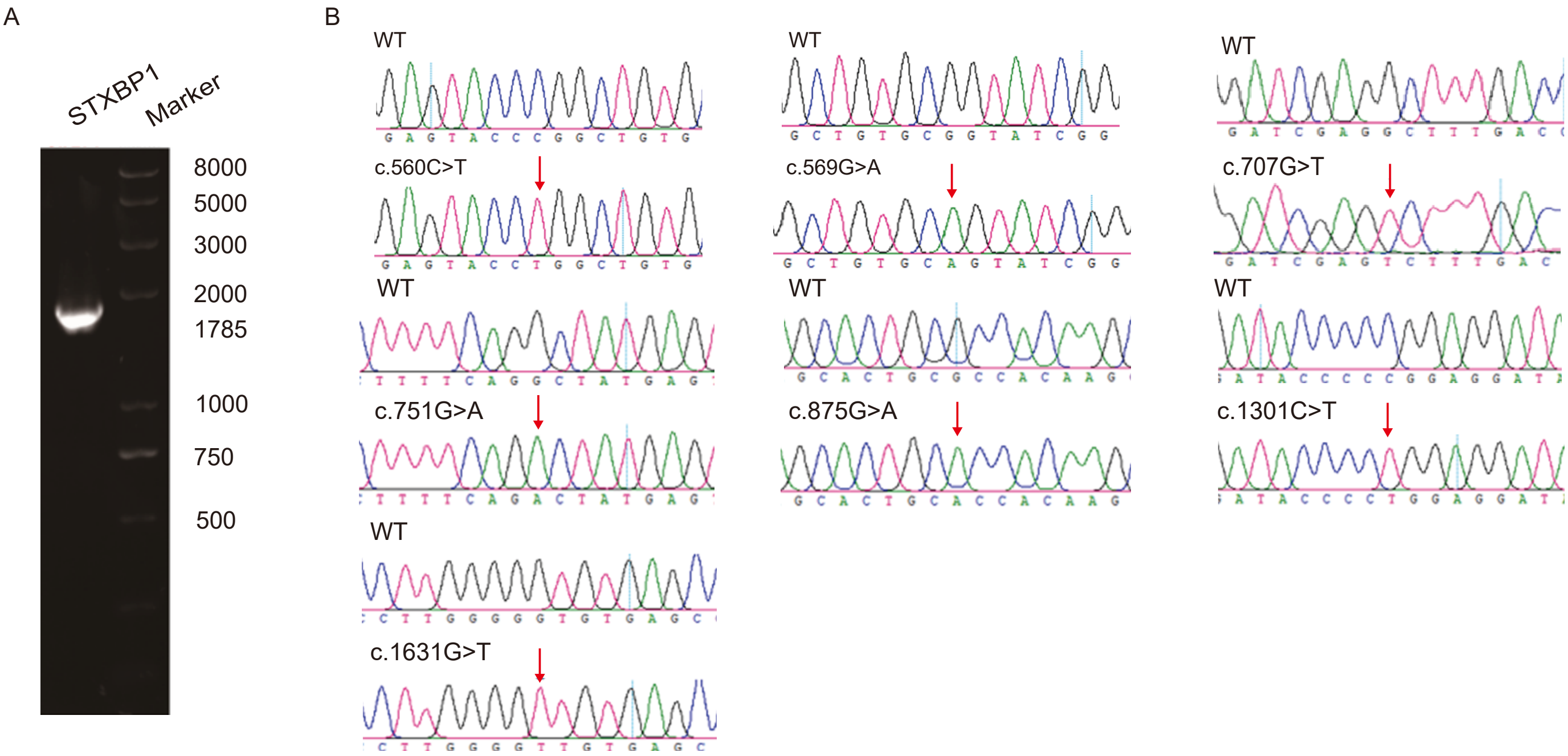

A
STXBP1
Marker
8000
5000
3000
2000
1785
1000
750
500
B
WT
c.560C>T
WT
c.569G>A
WT
c.707G>T
WT
c.751G>A
WT
c.875G>A
WT
c.1301C>T
WT
c.1631G>T
