# Supplementary material for: STXBP1 Variants Associated With Epilepsy With Variable Severity
Source: CNS Neurosci Ther. 2026 Aug 17;32(8):e71093. doi: 10.1002/cns.71093 (PMC13482176; doi:10.1002/cns.71093)
Supplement: Supplementary file 2 [file CNS-32-e71093-s001.docx]

**Table S2:** **Evaluation of previously reported *STXBP1* variants using the proposed scoring framework.**

| **Variant** | **Phenotype** | **Domain** | **DDG** | **Hydrogen bonds** | **Hydrophobicity** | **RSA** | **Expression** | **Total score** |
| --- | --- | --- | --- | --- | --- | --- | --- | --- |
| Val84Asp | DEE | + | + | + | + | + | + | 6+ |
| Cys180Tyr | DEE | - | + | - | + | + | + | 4+ |
| Met443Arg | DEE | - | + | + | + | + | + | 5+ |
| Leu446Phe | Asx | - | + | - | - | + | + | 3+ |
| Gly544Asp | DEE | - | + | + | + | + | + | 5+ |

DEE: developmental and epileptic encephalopathy, Asx: asymptomatic.
