# Supplementary material for: STXBP1 Variants Associated With Epilepsy With Variable Severity
Source: CNS Neurosci Ther. 2026 Aug 17;32(8):e71093. doi: 10.1002/cns.71093 (PMC13482176; doi:10.1002/cns.71093)
Supplement: Supplementary file 3 [file CNS-32-e71093-s002.docx]

**Table S1. Missense variants were introduced into wild-type *STXBP1* vectors by PCR-based site-directed mutagenesis.**

| **Mutation sites** | **Primer sequence** |
| --- | --- |
| STX (p.Pro187Leu/c.560C>T) | F: CCCTGAAGGAGTACCTGGCTGTGCGGTATC  R: GATACCGCACAGCCAGGTACTCCTTCAGGG |
| STX (p.Arg190Gln/ c.569G>A） | F: AGTACCCGGCTGTGCAGTATCGGGGGGAAT  R: ATTCCCCCCGATACTGCACAGCCGGGTACT |
| STX (p.Gly236Val/ c.707G>T) | F: TGATCCTGGATCGAGTCTTTGACCCCAGCT  R: AGCTGGGGTCAAAGACTCGATCCAGGATCA |
| STX (p.Ala251Thr/c.751G>A) | F: GAATTGACTTTTCAGACTATGAGTTATGAT  R: ATCATAACTCATAGTCTGAAAAGTCAATTC |
| STX (p.Arg292His/ c.875G>A) | F: TGTGGATAGCACTGCACCACAAGCACATCG  R: CGATGTGCTTGTGGTGCAGTGCTATCCACA |
| STX (p.Pro434Leu/ c.1301C>T) | F: ACGCCCAGATACCCCTGGAGGATAGTGAGA  R: TCTCACTATCCTCCAGGGGTATCTGGGCGT |
| STX (p.Gly544Val/ c.1631G>T) | F: TTTTCATCCTTGGGGTTGTGAGCCTGAATG  R: CATTCAGGCTCACAACCCCAAGGATGAAAA |
